# Emergence of *Plasmodium malariae* and Co-endemicity of 3 *Plasmodium* Species Parasites, Khanh Hoa Province, Vietnam, 2023–2025

**DOI:** 10.3201/eid3210.260751

**Published:** 2026-10

**Authors:** Tuấn Cường Võ, Chau Van Khanh, Hương Giang Lê, Nguyen Cong Trung Dung, Đăng Thùy Dương Nguyễn, Thu Hằng Nguyễn, Byoung-Kuk Na, Huynh Hong Quang

**Affiliations:** Gyeongsang National University College of Medicine and Institute of Health Science, Jinju, South Korea (T.C. Võ, H.G. Lê, D.T.D. Nguyễn, T.H. Nguyễn, B.K. Na); Institute of Malariology, Parasitology, and Entomology, Quy Nhon, Vietnam (C.V. Khanh, N.C.T. Dung, H.H. Quang)

**Keywords:** Malaria, Plasmodium malariae, Plasmodium falciparum, Plasmodium vivax, parasites, vector-borne infections, co-endemicity, Vietnam

## Abstract

A large malaria outbreak caused by *Plasmodium malariae* parasites occurred in Khanh Hoa Province, Vietnam, in 2023. Since then, *P. malariae* parasites have persisted in the region alongside *P. falciparum* and *P. vivax* species, changing malaria patterns. Those findings reinforce the need for strengthened surveillance in the region.

Malaria, a mosquitoborne disease caused by *Plasmodium* species parasites, poses a global health burden. Malaria incidence and mortality rates have markedly decreased in Vietnam over the past few decades (*1*); however, the Central Highlands region is a malaria hotspot, accounting for >90% of malaria cases in the country. *P. falciparum* and *P. vivax* are the major parasite species responsible for malaria in the region (*2*,*3*), whereas *P. malariae* cases historically have been rare. However, a large *P. malariae* outbreak occurred in Khanh Hoa Province, Vietnam, in 2023 (*4*), and the epidemic has persisted, further complicating malaria patterns and control efforts in the province. We investigated the epidemiologic characteristics of malaria cases and *Plasmodium* species in Khanh Hoa Province, Vietnam, during January 2023–December 2025, to characterize the epidemic in the province.

## The Study

We retrieved epidemiologic data on malaria cases from the Electronic Case-based Disease Surveillance—Malaria and Mosquito-borne Diseases Reporting System, a national electronic reporting system in Vietnam. We performed statistical analyses by using negative binomial regression followed by Tukey-adjusted pairwise comparisons. We calculated incidence rate ratios (IRRs) and 95% CIs and considered p<0.05 statistically significant.

During the 3 years studied, we identified a total of 493 symptomatic malaria cases in the province that were confirmed by standard microscopic examination and molecular methods targeting parasite 18S rRNA (*5*). Before 2023, the region had few reported malaria cases annually, just 3 in 2021 and 12 in 2022. However, cases increased sharply in 2023 and peaked in July 2023, when *P. malariae* emerged, gradually declining thereafter (Figure 1, panel A). 

**Figure 1:**
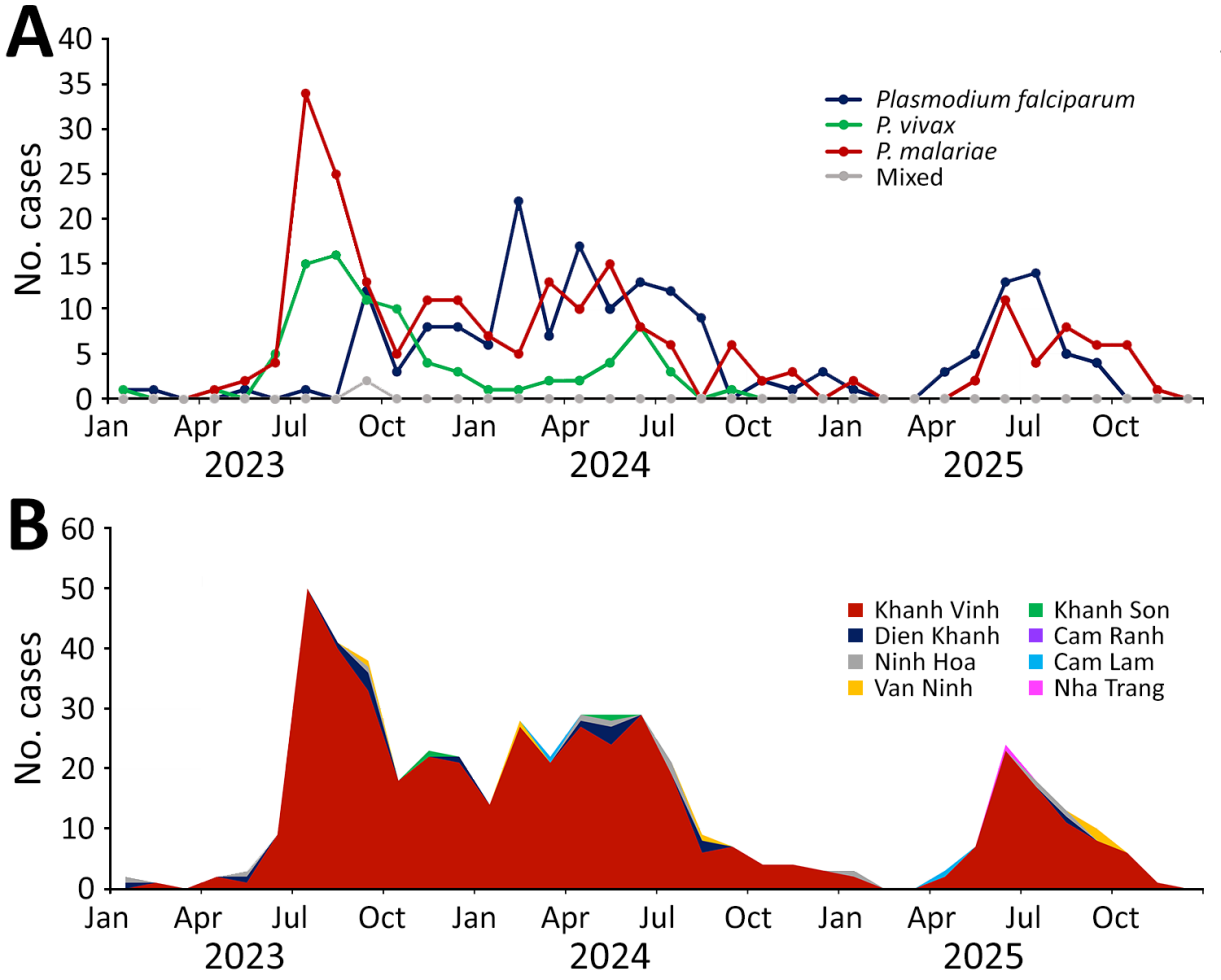
Malaria incidences from a study of emergence of *Plasmodium malariae* and co-endemicity of 3 *Plasmodium* species parasites, Khanh Hoa Province, Vietnam, 2023–2025. A) Monthly incidences by *Plasmodium* species; B) geographic distribution in the province.

Annual malaria cases exhibited typical summer peaks and totaled 209 in 2023, 199 in 2024, and 85 in 2025. During those years, most malaria cases were caused by *P. malariae* (44.8%; n = 221) and *P. falciparum* (36.9%; n = 182), whereas *P. vivax* (17.9%; n = 88) and mixed species (0.4%; n = 2) infections occurred less frequently. Three *Plasmodium* species were co-endemic in 2023 and 2024, but only *P. malariae* and *P. falciparum* were prevalent in 2025. Of the 8 districts and cities in Khanh Hoa Province, Khanh Vinh district had the most (93.1%; n = 459) cumulative malaria cases, whereas the other areas had <15 cases each, fluctuating from 0 in Cam Ranh to 14 in Dien Khanh (Figure 1, panel B). *P. malariae* is reportedly common in mixed co-infections with other species, particularly *P. falciparum* (*6*). However, molecular methods confirmed that all *P. malariae* cases in Khanh Hoa Province were monoinfections.

We further investigated the temporal and spatial dynamics of malaria cases in Khanh Vinh district (Figure 2). In 2023, *P. malariae* was most prevalent (104 cases), followed by *P. vivax* (62 cases) and *P. falciparum* (29 cases). Geographic overlap of the 3 species occurred in 6 communes, including Khanh Thuong and Lien Sang, but Khanh Dong, Khanh Phu, Cau Ba, and Khanh Nam had only 1 *Plasmodium* species identified, either *P. falciparum* or *P. malariae*. In 2024, species’ geographic distributions changed; *P. falciparu*m cases increased in Khanh Dong (n = 53) and Khanh Phu (n = 23), and distribution expanded to other communes. At the same time, overall *P. malariae* cases decreased to 72 (IRR 1.444 [95% CI 0.750–2.782]; p = 0.272), particularly in Son Thai (from 34 in 2023 to 3 in 2024), but no overall distribution changes were evident. Most (n = 40) *P. malariae* cases were detected in Khanh Thuong, but cases were found in most communes, except Khanh Dong and Khanh Trung. Meanwhile, *P. vivax* cases markedly decreased (n = 22; IRR 2.818 [95% CI 2.156–3.685]; p<0.001) in 2024 and had a narrower geographic distribution; most (n = 17) cases were reported in Khanh Thuong. We observed overlap of the 3 species in 3 communes, most notably in Khanh Thuong (Figure 2). In 2025, total malaria cases in Khanh Vinh district declined to 77, and no *P. vivax* cases were detected in the district. However, *P. falciparum* (n = 41) and *P. malariae* (n = 36) still exhibited geographic overlap in 5 communes. Those 3-year epidemiologic data suggested that overall malaria incidence in Khanh Vinh district decreased gradually and that endemic areas also contracted over time. However, *P. malariae* endemicity persists with other *Plasmodium* species.

**Figure 2:**
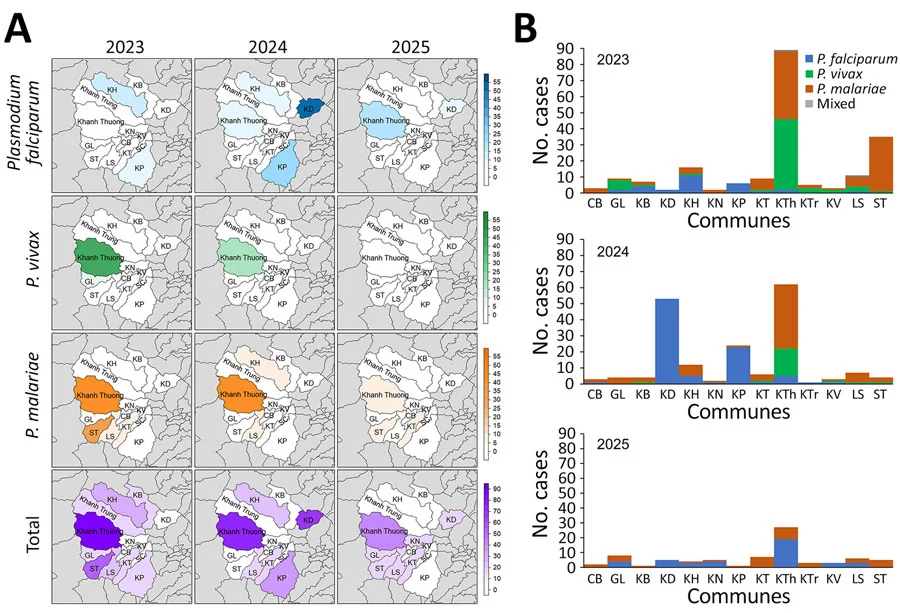
Changing pattern of malaria from a study of emergence of *Plasmodium malariae* and co-endemicity of 3 *Plasmodium* species parasites, Khanh Hoa Province, Vietnam, 2023–2025. A) Geographic distribution of *Plasmodium* species over time; B) annual malaria cases by commune and *Plasmodium* species. CB, Cau Ba; GL, Giang Ly; KB, Khanh Binh; KD, Khanh Dong; KH, Khanh Hiep; KN, Khanh Nam; KP, Khanh Phu; KT, Khanh Thanh; Kth, Khanh Thuong; KTr, Khanh Trung; KV, Khanh Vinh Town; LS, Lien Sang; SC, Song Cau; ST, Son Thai.

Among malaria patients in Khanh Vinh district, 338 were male and 121 were female, a 2.8:1 ratio (Figure 3, panel A), and most (n = 344; 75.0%) were >15 years of age (Figure 3, panel B). Ethnic analysis revealed that the Raglai community was the most affected (304 cases; 66.2%), followed by the Kinh (53 cases; 11.6%) and Co Ho (50 cases; 10.9%) populations (Figure 4, panel A). Affected persons largely were forest-goers (n = 314; 68.4%) but also included rangers (n = 29; 6.3%) and government or factory workers (n = 15; 3.3%) (Figure 4, panel B). Heavy dependence on the forest or forest farming for subsistence places the Raglai population at greater risk for malaria infection, and that community accounts for most malaria cases in Central Vietnam (*7*,*8*). We observed no meaningful association between patient demographic characteristics and specific *Plasmodium* species infections.

**Figure 3:**
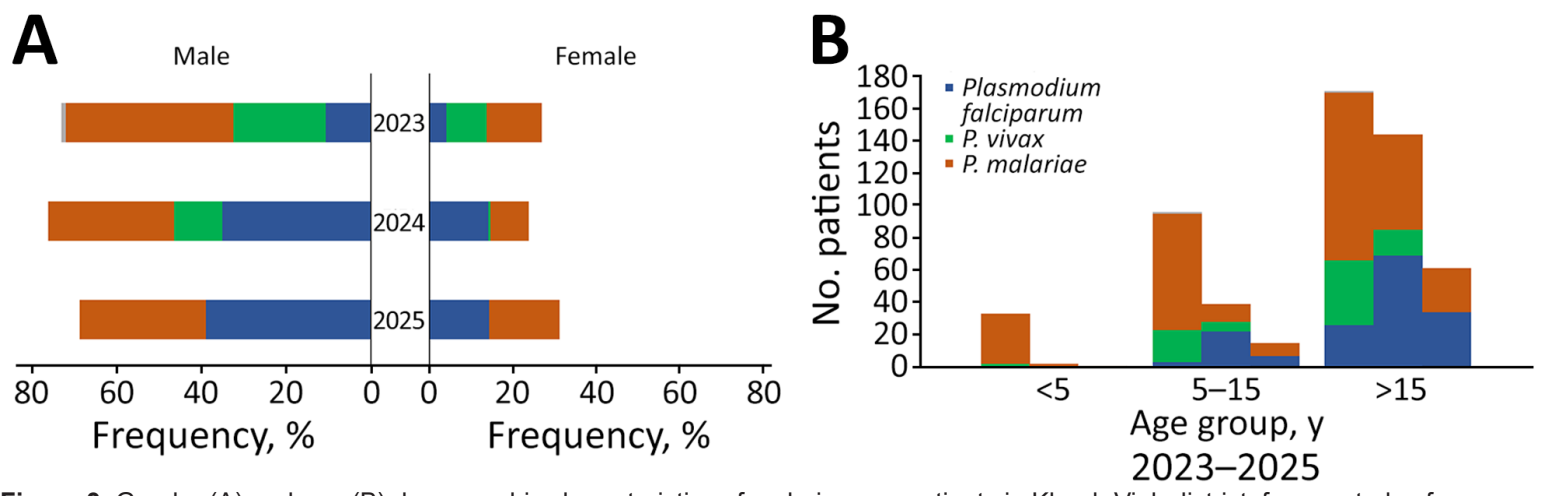
Gender (A) and age (B) demographic characteristics of malaria case-patients in Khanh Vinh district, from a study of emergence of *Plasmodium malariae* and co-endemicity of 3 *Plasmodium* species parasites, Khanh Hoa Province, Vietnam, 2023–2025.

**Figure 4:**
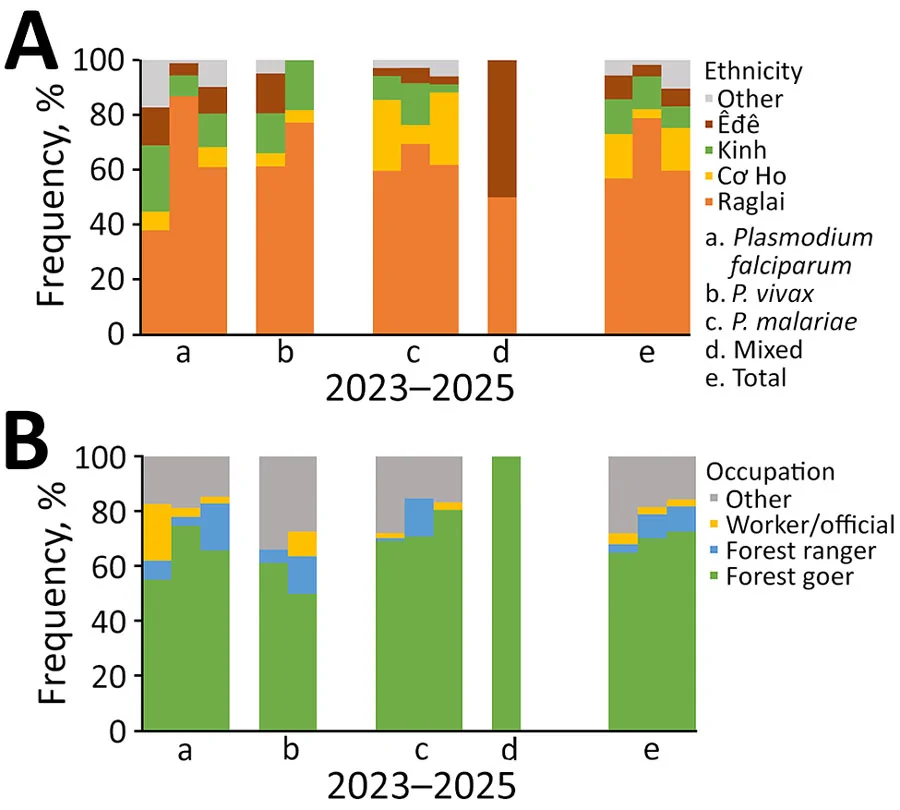
Ethnicity (A) and occupation (B) of malaria case-patients in Khanh Vinh district, from a study of emergence of *Plasmodium malariae* and co-endemicity of 3 *Plasmodium* species parasites, Khanh Hoa Province, Vietnam, 2023–2025.

The cause of the *P. malariae* outbreak in Khanh Hoa Province, particularly in Khanh Vinh district, remains unclear. Successful interventions targeting *P. falciparum* and *P. vivax* parasites might have inadvertently expanded the ecologic niche of *P. malariae* (*9*). Given the increase in *P. malariae* infections (10 of 12 total malaria cases) in Khanh Hoa Province in 2022, *P. malariae* might have begun to emerge at that time. Persistent asymptomatic infections in the province might also have contributed to the outbreak. Indeed, high rates of asymptomatic and submicroscopic *P. falciparum* and *P. vivax* infections have been identified in Central Vietnam (*10*,*11*). Those hypotheses are supported by our pilot study on asymptomatic malaria in Khanh Hoa Province (T.C. Võ et al., unpub. data), in which 1.57% of 1,401 residual blood samples collected from 6 communes of Khanh Vinh district during January 2024–April 2025 demonstrated asymptomatic and submicroscopic *P. falciparum* and *P. malariae* infections. The COVID-19 pandemic might also have contributed to that outbreak by straining or fragmenting the health infrastructure, as reported in other countries in Southeast Asia (*12*,*13*).

Genetic analysis of *P. malariae* parasites in Khanh Hoa Province showed notable genetic variations in major polymorphic markers, such as apical membrane antigen 1 and circumsporozoite surface protein (*14*; T.C. Võ et al., unpub. data), despite the homogeneity of the parasite population collected in a limited region over a short period. Although molecular markers of antimalarial drug resistance have not yet been confirmed in *P. malariae* parasites (*15*), molecular analyses of orthologous genes associated with drug resistance in *P. falciparum* and *P. vivax* parasites from Khanh Hoa Province revealed no remarkable mutations associated with antimalarial drug resistance (T.C. Võ et al., unpub. data), which is consistent with no identified clinical drug failure to date in the region. Further genetic studies are underway to elucidate the population structure of *P. malariae* parasites in Khanh Hoa Province and to examine their genetic nature. Studies on mosquito vectors and environmental changes associated with malaria transmission dynamics are also underway.

## Conclusions

Co-endemicity of 3 *Plasmodium* species after a recent *P. malariae* outbreak has altered malaria endemicity patterns in Khanh Hoa Province, Vietnam. As of 2025, *P. malariae* cases mainly occurred in Khanh Vinh district, and the epidemic appears to be contained. However, that *P. malariae* outbreak changed the overall malaria epidemic pattern in the province and resulted in the persist, unstable co-endemicity of multiple *Plasmodium* species, suggesting the need for strengthened surveillance and control interventions.
